# New insights into the pathogenesis of leprosy: contribution of subversion of host cell metabolism to bacterial persistence, disease progression, and transmission

**DOI:** 10.12688/f1000research.21383.1

**Published:** 2020-01-31

**Authors:** Cristiana Santos de Macedo, Flavio Alves Lara, Roberta Olmo Pinheiro, Veronica Schmitz, Marcia de Berrêdo-Pinho, Geraldo Moura Pereira, Maria Cristina Vidal Pessolani

**Affiliations:** 1 Center for Technological Development in Health (CDTS), Oswaldo Cruz Foundation (FIOCRUZ), Rio de Janeiro, 21040-361, Brazil; 2 Laboratory of Cellular Microbiology, Oswaldo Cruz Institute (IOC), Oswaldo Cruz Foundation (FIOCRUZ), Rio de Janeiro, 21040-360, Brazil; 3 Leprosy Laboratory, Oswaldo Cruz Institute (IOC), Oswaldo Cruz Foundation (FIOCRUZ), Rio de Janeiro, 21040-360, Brazil

**Keywords:** Leprosy, disease tolerance, metabolism, immune response, regulatory T cells, lipids, mycobacterium

## Abstract

Chronic infection by the obligate intracellular pathogen *Mycobacterium leprae* may lead to the development of leprosy. Of note, in the lepromatous clinical form of the disease, failure of the immune system to constrain infection allows the pathogen to reproduce to very high numbers with minimal clinical signs, favoring transmission. The bacillus can modulate cellular metabolism to support its survival, and these changes directly influence immune responses, leading to host tolerance, permanent disease, and dissemination. Among the metabolic changes, upregulation of cholesterol, phospholipids, and fatty acid biosynthesis is particularly important, as it leads to lipid accumulation in the host cells (macrophages and Schwann cells) in the form of lipid droplets, which are sites of polyunsaturated fatty acid–derived lipid mediator biosynthesis that modulate the inflammatory and immune responses. In Schwann cells, energy metabolism is also subverted to support a lipogenic environment. Furthermore, effects on tryptophan and iron metabolisms favor pathogen survival with moderate tissue damage. This review discusses the implications of metabolic changes on the course of *M. leprae* infection and host immune response and emphasizes the induction of regulatory T cells, which may play a pivotal role in immune modulation in leprosy.

## Introduction

In recent years, the literature has provided extensive evidence indicating the crosstalk between cellular metabolism and inflammatory/immune responses and how they may influence each other ^1– 3^. In parallel, the concept of disease tolerance that decreases immunopathology caused by pathogens or the immune responses against them has been proposed ^4^. The major point discussed in this article is how *Mycobacterium leprae* host cell metabolism subversion contributes to bacterial persistence and disease tolerance in leprosy, pointing to regulatory T (Treg) cells as potential key players in this scenario. Leprosy constitutes an excellent model for understanding the mechanisms of the immune system regulation that can be applied to other chronic inflammatory diseases in humans.

Leprosy is a chronic infectious disease that affects the skin and peripheral nervous system and is caused by the intracellular pathogen *M. leprae*. Peripheral nerve injury is the most severe symptom affecting patients with leprosy. Nerve impairment may become irreversible when the diagnosis is late, resulting in permanent disability and physical deformities, which are hallmarks of the disease. Although leprosy is a treatable disease, it is still endemic in some countries, such as India and Brazil ^5^. Three decades ago, with the implementation of multidrug therapy (MDT) by the World Health Organization, patients were allowed to receive treatment free of charge and the caseload has dropped dramatically from 10 to 12 million in the mid-1980s ^6^ to the current 200,000 cases per year. However, the detection rate of new cases has remained constant, indicating little impact on leprosy transmission ^5^.

Compared with other bacterial pathogens, *M. leprae* displays a very low genetic variability ^7^, allowing researchers to trace the dissemination of the disease by the great human migratory movements around the world ^8^. A recent phylogenetic study indicated that the most ancient strains infecting humans are typical of East Asia ^9^. *M. lepromatosis*, a bacterium that shares about 87% genome homology with *M. leprae* ^10^, was recently identified as the causative agent of a diffuse clinical form of leprosy known as “pretty leprosy” ^11^. *M. leprae* was initially thought to be restricted to humans, but armadillos in the US ^12^, red squirrels in the UK ^13^, and non-human primates in Africa ^14^ have been identified as natural reservoirs of this bacillus.

The spectrum of clinical forms of leprosy includes at one extremity the polar tuberculoid leprosy, which features low bacillary load, positive lepromin skin test, *in vitro* lymphoproliferative response to *M. leprae* antigens, and interferon gamma (IFN-γ) production in response to the bacillus. There are also intermediate forms of the disease, and at the other extreme of the spectrum is the polar lepromatous form, which features high bacillary load, negative skin response to lepromin, and low to undetectable T-cell proliferation and IFN-γ levels when blood mononuclear cells of these patients are cultured in the presence of *M. leprae* antigens ^15, 16^. Patients with lepromatous leprosy are considered the main source of *M*. *leprae* transmission, and their early diagnosis and treatment are mandatory in leprosy control programs. Despite the high bacterial burden, patients with lepromatous leprosy display moderate pathology and are a relevant model to study disease tolerance in humans.

The absence of an experimental model and the impossibility of the pathogen to grow *in vitro* have delayed the understanding of leprosy pathogenesis. The studies are limited to *in vitro* assays exploring early stages of host–pathogen interaction and analysis of patients’ clinical specimens, such as skin, nerve, and blood. In this article, we describe *in vitro* studies that indicate the capacity of *M. leprae* to modulate the metabolism of its main cell targets, Schwann cells and macrophages, and how these changes may favor bacterial persistence with impact on the immune response to infection, such as the generation of Treg cells. Moreover, we discuss complementary *in vivo* studies, which with the help of powerful new technologies are reinforcing the idea of the potential contribution of these metabolic effects to the scenario observed in lepromatous leprosy, where high bacterial burden associated with disease tolerance promotes disease perpetuation at the population level.

## *Mycobacterium leprae* infection induces drastic changes in host cell metabolism

The ability of *M. leprae* to chemically and metabolically alter the cytosol environment of the host cell was first described by Rudolf Virchow (1821–1902) in the late nineteenth century ^17^. The German physician, pathologist, and microbiologist observed that macrophages from skin lesions of patients with lepromatous leprosy had a foamy appearance, referred to as Lepra or Virchow cells. Later on, this phenomenon was also observed in Schwann cells present in nerves from patients with leprosy ^18^. Initial histochemical analysis of the lipids in human leprosy revealed the accumulation of both fatty acids and phospholipids in lepromatous lesions ^19^. More recently, accumulation of oxidized phospholipids and cholesterol/cholesterol esters was also demonstrated ^20, 21^. *In vitro* studies confirmed the capacity of *M. leprae* to induce lipid accumulation in infected cells in the form of lipid droplets, which are storage organelles of the cell. This induction is triggered by Toll-like receptor 6 (TLR6) (for both cells) and TLR2 (essential only for macrophage induction)-mediated signals ^22, 23^. Lipid droplets were shown to migrate to bacterium-containing phagosomes and inhibition of this event enhances bacterial killing ^22– 24^. A similar phenomenon was observed in *M. tuberculosis*–infected macrophages, where infection-induced lipid accumulation is critical to the success of the infection and resistance against antibiotics ^25, 26^.

A shift of the infected cell to a lipogenic phenotype implies drastic changes in the host cell metabolism. Indeed, a global gene expression analysis of leprosy skin lesions revealed higher expression of host lipid metabolism genes in lepromatous lesions ^20^. In 2014, histochemical, metabolomics, and transcription analysis confirmed the increase in exogenous lipid uptake, *de novo* biosynthetic pathways, and lipid degradation pathways in these lesions ^21^. Moreover, sterol regulatory element-binding proteins (SREBPs) and peroxisome proliferator-activated receptor gamma (PPAR-γ), master transcriptional factors involved in lipid metabolism regulation, were shown to be upregulated in *M. leprae* infection ^21, 27^. Also, imaging mass spectrometry of skin lesions of multibacillary patients before and after MDT showed an upregulation of phospholipid metabolism in the dermis, which reverted to normal patterns after MDT ^28^.

In the context of Schwann cells, it was also shown that *M. leprae* alters host cell energy metabolism at several points, which favors lipid accumulation ^29^. A major effect is the decrease of the mitochondrial action potential, which generates several adaptive advantages to *M. leprae*, such as drop in beta oxidation, resulting in an exacerbation of lipid accumulation in the host cell cytosol; reduction in the generation of reactive oxygen species, which is specially threatening in a lipid-rich environment; and lowering in the reducing power consumption from glucose oxidation. All this accumulated reducing power, which was previously consumed for ATP generation, is now available for maintenance of the glutathione-based antioxidant system and lipid biosynthesis in the infected cells ^29^.

The activity of mitochondrial electron transport chain, the main ATP cell source, is reduced in the infected cell, and ATP production becomes more dependent on glycolysis, which generates much less ATP in comparison with oxidative phosphorilation. Thus, to maintain cytoplasmic ATP levels, infected Schwann cells show increased glucose uptake, with a concomitant and significant increase in the pentose phosphate pathway ^29^. Glucose uptake is positively modulated by insulin growth factor 1 (IGF-1) signaling, also upregulated by the bacillus ^30^. Thus, *M. leprae* alters Schwann cell metabolism to allocate as much carbon and reducing power as possible to lipid synthesis.

Lipid droplets represent a link between energy metabolism and innate immune response. These organelles are sites for polyunsaturated fatty acid (PUFA)-derived lipid mediator biosynthesis ^31^. Cyclooxigenase-2 (COX-2), responsible for the biosynthesis of prostaglandins, was detected in association with lipid droplets in *M. leprae* infection ^22, 32, 33^. Indeed, metabonomics studies using Fourier transform mass spectrometry showed that PUFA metabolism is upregulated during leprosy in blood and skin ^34, 35^. Analysis of serum samples showed an upregulation of omega-3 and omega-6 PUFA metabolism and the presence of higher levels of omega-6–derived—prostaglandin E _2_ (PGE _2_) and lipoxin A _4_ (LXA _4_)—and omega-3 (resolvin D1-RvD1)-derived lipid mediators, especially in lepromatous patients with high bacterial load ^34, 36^. Omega-3 PUFAs such as eicosapentaenoic acid (EPA) and docosahexaenoic acid (DHA) present direct anti-inflammatory effects through interaction with free fatty acid receptor 4 (FFAR4/GPR120), which leads to the blockage of both nuclear factor kappa B (NF-κB)-mediated inflammatory responses and NLRP3 inflammasome activation. They also favor macrophage polarization to alternatively activated macrophages (M2) anti-inflammatory phenotype and the proliferation of Treg cells (reviewed in 37, 38). The anti-inflammatory properties of omega-3 PUFAs have been shown to be mediated, at least in part, by a new family of pro-resolving lipid mediators that include resolvins, protectins, and maresins ^39^: these compounds at the same time favor Treg cell generation and function and inhibit T helper 1 (Th1) and Th17 differentiation through GPR32 and GPR32-ALX/FPR2 receptors, respectively ^40^.

*In vitro* assays showed that lipid droplet accumulation and the innate immune response are very closely related in *M. leprae*–infected cells. Inhibition of lipid droplet formation induces a switch from an anti-inflammatory to a pro-inflammatory profile ^22^. PGE _2_ is abundantly produced by infected Schwann cells and macrophages, and this production is dependent of lipid droplet accumulation ^22, 23^. PGE _2_ is a potent immune modulator that promotes Treg cell accumulation and inhibits Th1 and macrophage microbicidal functions ^41^, limiting cellular immune responses that could control *M. leprae* infection. Also, inhibition of either PGE _2_ production or lipid droplet formation resulted in downmodulation of production of interleukin-10 (IL-10) ^23^, a potent anti-inflammatory mediator and inhibitor of Th1 response ^42^.

*M. leprae*–infected macrophages present in skin lesions of patients with lepromatous leprosy have been shown to be good producers of PGE _2_ and IL-10 ^32, 43^. These cells display several markers of M2 as explored in a recent review ^44^. Particularly, IL-10 has been pointed to play a pivotal role in the permissive phenotype of lepromatous macrophages by inducing highly phagocytic activity concomitant with the impairment of antimicrobial pathways allowing the pathogen to reach high numbers in these cells ^43^. Probably multiple pathways are implicated in the induction of IL-10 in *M. leprae*–infected macrophages *in vivo*. As mentioned earlier, PGE _2_ induced by *M. leprae* through lipid droplet formation influences the levels of IL-10 production in *in vitro* infected cells ^22^. The potential role of PGE _2_ is reinforced by evidence that it induces macrophage IL-10 production ^45^ and augments its signaling and function ^46^. Type I IFN and IL-27 have also been implicated in increased IL-10 expression in lepromatous cells ^47, 48^. Taken together, all of these observations indicate that *M. leprae*–induced lipid modulation has important pathophysiological consequences for bacterial survival and proliferation with a clear demonstration of the activation of disease tolerance mechanisms that allow the perpetuation of the infection.

Another metabolic pathway modulated by *M. leprae* with a potential strong contribution to the high bacterial burden associated with low immunopathology observed in lepromatous leprosy is the tryptophan degradation pathway. Indoleamine 2,3-dioxygenase (IDO-1) was shown to be highly expressed in macrophages and dendritic cells of lepromatous lesions. Moreover, higher activity of the enzyme was detected in the serum of patients with lepromatous leprosy compared with that of patients with the tuberculoid form ^49^. IDO-1 catalyzes the first and rate-limiting step of tryptophan degradation that results in tryptophan depletion and generation of bioactive catabolites known as kynurenins. Although activation of this pathway can directly promote the killing of some pathogens, a major biological role of IDO-1 is the suppression of innate and adaptive immunity by multiple mechanisms ^50, 51^. One of them refers to the capacity of kynerunin to induce Treg cell differentiation by signaling through the aryl hydrocarbon receptor (AhR) ^52^. In cancer models, it was observed that IDO-1 and COX-2 activities are directly related, as PGE _2_ strongly induces *IDO1* transcription ^53^.

Lastly, iron (Fe) metabolism regulation may also contribute to the scenario of low pathogen resistance associated with disease tolerance observed in lepromatous leprosy. In comparison with tuberculoid lesions, *M. leprae*–infected macrophages in lepromatous lesions show abundant Fe deposits as ferritin, suggesting that this essential nutrient is fully available for bacterial intracellular growth ^54^. This is reinforced by the high content of Fe found in *in vivo*–grown *M. leprae* bacterioferritin ^55^. Moreover, high intracellular Fe levels may contribute to the defective capacity of *M. leprae*–infected macrophages to respond to activating signals such as IFN-γ signaling ^56^, as demonstrated in previous studies ^57, 58^. The high Fe levels in lepromatous lesions can be explained by an imbalance of the hepcidin/ferroportin-1 (FPN) axis detected in these patients. FPN is the only known Fe cellular exporter protein and its level is downregulated by hepcidin, an acute phase protein ^59^. Patients with lepromatous leprosy show higher expression levels of hepcidin ^60^ and lower levels of FPN ^54^. Additionally, a higher ratio of transferrin receptor/FPN, higher levels of CD163 (a scavenger receptor that binds to the hemoglobin-haptoglobin complex), and higher levels of both light and heavy chains of ferritin were observed in lepromatous lesions contributing to Fe accumulation ^54^.

**Figure 1.**
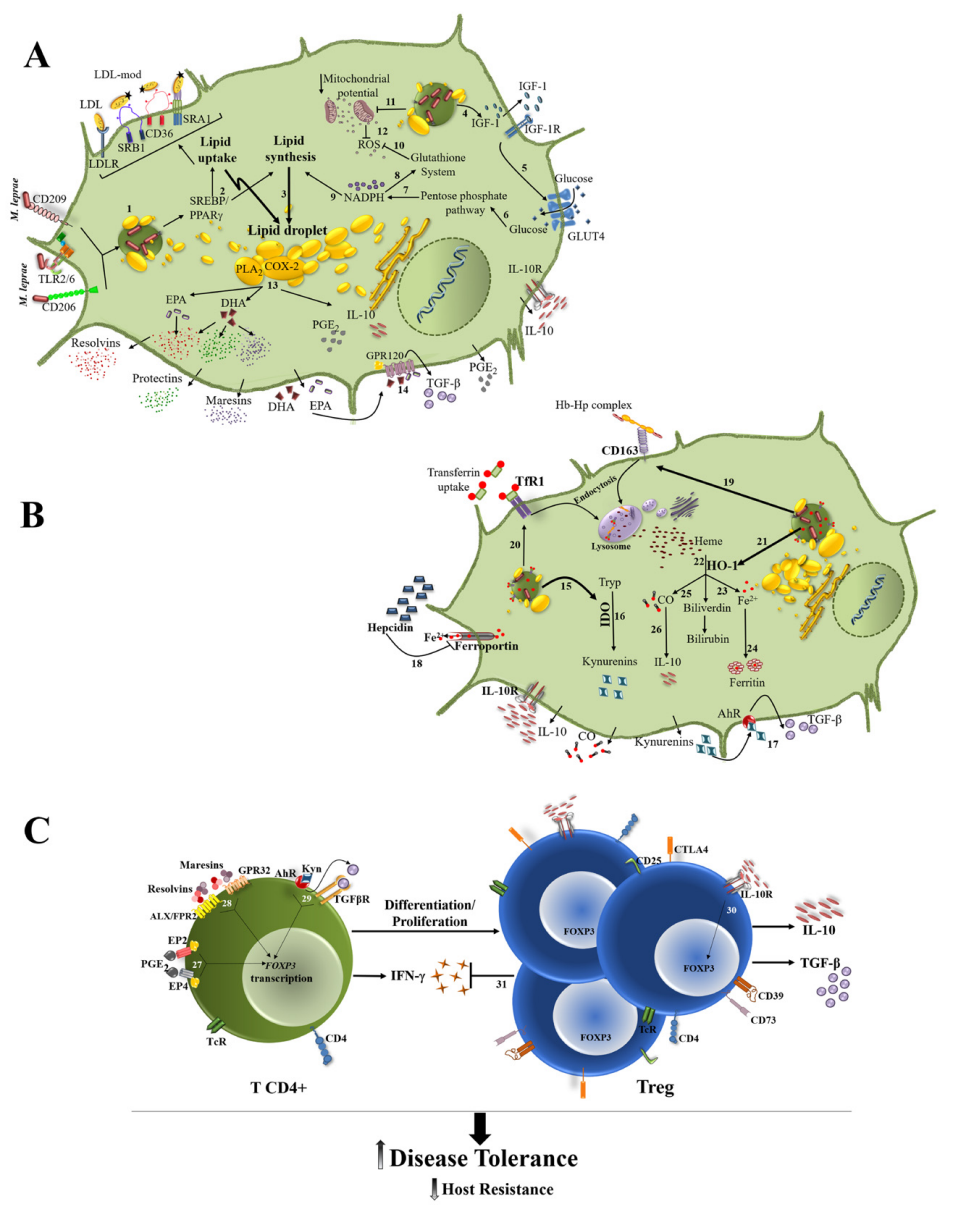
*Mycobacterium leprae* subversion of host cell metabolism and immunomodulation in lepromatous leprosy. Effects on lipid and energy ( **A**) and on iron and tryptophan ( **B**) metabolisms are shown separately to make the scheme clearer. (1) *M. leprae* binding to host cell surface receptors, such as CD206, CD209, and TLR2/6, leads to bacterial internalization and activation of signaling cascades that result in the expression and activation of SREBP and PPARγ. (2, 3) SREBP and PPARγ upregulate the expression of LDLR, CD36, SRA1, and SRB1 bringing lipids into the cell, and of proteins involved in *de novo* lipid biosynthesis, resulting in intracellular lipid accumulation as lipid droplets. (4, 5) *M. leprae* induces IGF-1 production, which may be responsible for the higher glucose uptake in infected cells. (6–10) Glucose is shunted to the pentose pathway for the generation of the NADPH necessary for lipid synthesis and for maintenance of the glutathione-based antioxidant system. (11, 12) The infection also decreases the mitochondrial membrane potential, reducing the generation of ROS production. (13) COX-2 and PLA _2_ present in lipid droplets upregulate the omega-3 and omega-6 PUFA (EPA and DHA) metabolism, increasing the formation of anti-inflammatory/pro-resolving lipid mediators, such as resolvins, protectins, maresins, and PGE _2_, which is associated with IL-10 production. (14) EPA and DHA activate GPR120 leading TGF-β production. (15, 16) *M. leprae* elevates IDO that degrades Tryp to kynurenines products. (17) Kynurenines activate AhR leading to TGF-β production. (18) *M. leprae* infection raises the levels of hepcidin that degrades the iron exporter ferroportin 1, resulting in higher levels of intracellular iron. (19, 20) The infection also upregulates the expression of CD163 and TfR1, increasing the iron intracellular levels. (21, 22) The heme molecules are degraded by HO-1, overexpressed in lepromatous leprosy lesions. (23, 24) Iron is stored in the form of ferritin. (25–26) The generated CO can induce IL-10 production, which can act in a paracrine and autocrine way, enhancing IDO expression and activity. ( **C**) (27–29) The tissue microenvironment enriched in anti-inflammatory/pro-resolution moleculesactivates *FOXP3* transcription, favoring Treg cells differentiation and proliferation. (30) IL-10 potentiates Treg differentiation. (31) Treg cells downmodulate CD4 ^+^ T cell response, decreasing IFN-γ production and resistance to infection, allowing uncontrolled bacterial proliferation while promoting disease tolerance. AhR, aryl hydrocarbon receptor; ALX/FPR2, G-protein coupled formyl peptide receptor 2; CD, cluster of differentiation; CO, carbon monoxide; COX-2, cyclooxygenase-2; CTLA4, cytotoxic T-lymphocyte associated protein 4; DHA, docosahexaenoic acid; EP2 and EP4, prostaglandin E _2_ receptor 2 and 4; EPA, eicosapentaenoic acid; Fe, iron; FOXP3, forkhead box P3; GLUT4, glucose transporter 4; GPR32 and GPR120, G protein-coupled receptor 32 and 120; Hb, hemoglobin; HO-1, heme oxygenase 1; Hp, haptoglobin; IDO, indoleamine 2,3 dioxygenase; IFN-γ, interferon-gamma; IGF-1, insulin-like growth factor 1; IL-10, interleukine-10; LDL, low-density lipoprotein; NADPH, nicotinamide adenine dinucleotide 2′-phosphate; PGE _2_, prostaglandin E _2_; PLA _2_, phospholipase A _2_; PPARγ, peroxisome proliferator-activated receptor gamma; PUFA, polyunsaturated fatty acid; ROS, reactive oxygen species; SRA1 and SRB1, scavenger receptor A1 and B1; SREBP, sterol regulatory element-binding protein; TcR, T-cell receptor; TfR1, transferrin receptor 1; TGFβ, transforming growth factor beta; TLR2/6, Toll-like receptor 2/6; Treg, regulatory T cells; Tryp, tryptophan.

The same study also showed higher expression levels of heme oxygenase (HO-1) ^54^, suggesting extensive intracellular catabolism of heme with the generation of Fe, carbon monoxide, and bilirubin, which exert beneficial effects on tissue damage control ^4^. Mainly through the generation of carbon monoxide, several immunomodulatory effects have been attributed to HO-1, such as IL-10 induction, macrophage polarization to an M2-like phenotype ^59, 61^, and promotion of Treg cell differentiation and proliferation to the detriment of Th1 expansion ^62^. *M. leprae* was shown to increase IDO-1 expression and activity as well as CD163 expression in human monocytes/macrophages by IL-10 ^63, 64^. The addition of hemin to the cells increased *M. leprae* viability whereas treatment with an IDO inhibitor showed the opposite effect ^54^, reinforcing the idea that both heme and tryptophan metabolism are necessary for *M. leprae* survival inside human macrophages. Figure 1 summarizes the host cell metabolic pathways (discussed above) that are modulated during *M. leprae* infection and that culminate in the lepromatous form of the disease.

## Microenvironments, Treg cells, and leprosy

As discussed above, effects on cellular metabolism seem to create a microenvironment at the site of *M. leprae* infection that promotes enhanced Treg cell differentiation and activity ( Figure 1). Treg cells play a pivotal immunoregulatory role by controlling the intensity of the innate and adaptive immune responses in order to avoid immunopathology. In the context of leprosy, the generation of Treg cells may facilitate the progressive reduction of the host pathogen-specific IFN-γ response and resistance to infection and contribute to host disease tolerance.

At the population level, previous observations suggest that persistent exposure to *M. leprae* can induce negative modulation of IFN-γ response and effector function against *M. leprae* that, in some individuals, will allow the bacillary load to increase and lead to the onset of active disease. The evaluation of the *in vitro* production of IFN-γ by blood mononuclear cells in response to *M. leprae*–specific epitopes allowed the observation of a progressively reduced IFN-γ in individuals persistently exposed to *M. leprae* ^65^.

The downmodulation of T-cell response and IFN-γ production seen in more exposed asymptomatic individuals, and in patients with leprosy, is clearly linked to an increase in bacillary load across the spectrum of clinical forms. So a sequence of events, including microenvironments protected against effector mechanisms for initial survival of *M. leprae* in the host, *M. leprae*–triggered changes in infected cells, and tissues inducing an increase in the differentiation of T cells to a Treg cell phenotype, changing the ratio of pathogen-specific memory CD4-positive, FOXP3-negative cells (T CD4 ^+^) to memory Treg cells, generates conditions for a chronic infection. The analysis of the human T-cell repertoire in neonates and young adults using major histocompatibility (MHC)-peptide tetramers shows that, in the neonates, the ratio T CD4 ^+^:Treg cell is similar for self-antigens and epitopes of infectious agents. However, successful response to infection is followed by marked increase in the T CD4 ^+^:Treg cell ratio and this is due mostly to the higher number of T CD4 ^+^. In a murine model, evolution to chronic infection was linked to T CD4 ^+^:Treg cell ratios lower than in the healed animals ^66^. So evolution to chronic infection in leprosy requires conditions for the initial survival of *M. leprae* in the host, which will involve changes in the tissue microenvironment that will inhibit effector function against *M. leprae* and facilitate a reduction in the T CD4 ^+^:Treg cell ratio. Moreover, the predominance of Treg cells over T CD4 ^+^ will certainly contribute to the scenario of moderate immunopathology and host disease tolerance associated with the high bacterial burden in lepromatous leprosy.

The initial characterization of the CD4 ^+^CD25 ^+high^ as the subset with suppressor function in immune response followed by the discovery of forkhead box P3 (FOXP3), the lineage-specific transcription factor for this T-cell subset, provided critical tools for investigating the role of Treg cells in autoimmune diseases and infections ^67, 68^. Inhibition of IFN-γ production by tumor-infiltrating Treg cells, in murine experimental models of melanoma and adenocarcinoma, allowed activation of SREBP1 and fatty acid synthesis in immunosuppressive tumor-infiltrating macrophages ^69^. Helper T cells specific to cardiac myosin have been detected in a murine experimental model of myocardial infarction. These cells acquire *Foxp3* (murine gene nomenclature) expression and pro-healing functions following homing to the myocardium, once more demonstrating the impact of the microenvironment in the expression of regulatory function by T cells ^70^. An increase in the number of FOXP3 ^+^ T cells was observed in blood leukocytes of patients with lepromatous leprosy when compared with tuberculoid leprosy. The analysis of skin lesions also provided evidence of a higher frequency of FOXP3 ^+^ T cells in lepromatous leprosy ^71, 72^. Several studies using *FOXP3* expression in blood T cells and in skin lesions in leprosy for the identification of Treg cells have recently been published ^73– 79^. However, human memory T cells with no regulatory function can express *FOXP3* transiently following activation ^80^. Moreover, the investigations in leprosy, like those in other diseases and experimental models, need to take into account recent findings in the biology of Treg cells and standardized approaches for the characterization of Treg cell roles in leprosy ^81– 84^.

As the observed defect in T-cell responsiveness is restricted to *M. leprae* in leprosy, we can postulate the induction of memory T cells specific to *M. leprae* antigens with a reduction in the TCD4 ^+^:Treg cell ratio, in parallel with defective IFN-γ production and effector function against *M. leprae*, as the mechanism involved in the pathogenesis of leprosy and the different clinical forms of the disease. Peripheral Treg cells specific to infectious agents, and induced during infection, have been previously characterized ^66, 85, 86^. The investigation of these cells in the human immune response to *M. leprae* requires some additional steps to demonstrate the involvement of peripheral Treg cells specific to *M. leprae* antigens in leprosy. Human Treg cells are a heterogeneous group of cells. Transient expression of *FOXP3* occurs during activation of conventional human memory T cells ^80^. So it becomes a requirement in the characterization of these cells to demonstrate that the *FOXP3* gene has the epigenetic modifications necessary for its stable expression and that these Treg cells recognize *M. leprae* antigens. Another basic question to be addressed is how the Treg cells modulate *in vivo* the immune response to *M. leprae*. The *in vitro* mechanisms of negative modulation of immune response by antigen-specific Treg cells are not necessarily those relevant to inhibit immune response *in vivo* ^81^. Recent observations in murine experimental models demonstrate that removal of MHC-associated antigens in the antigen-presenting cells is an *in vivo* mechanism of action for inhibiting T-cell function in an antigen-specific way ^87^. Would this mechanism account for *M. leprae*–specific activity of Treg cells in leprosy and the defective immunity toward this bacillus seen in lepromatous leprosy?

The lack of experimental models for asymptomatic infection with *M. leprae* and leprosy is a major limitation for the identification of metabolic changes involved in the induction of Treg cells in the pathogenesis of the disease and in lepromatous leprosy. However, supernatants from dissociated cells, obtained from lepromatous leprosy lesions, could be a source of mediators for *in vitro* models of Treg cell differentiation and function, modulating effector mechanisms against *M. leprae*–infected cells ^40^. Moreover, mediators associated with *M. leprae*–infected macrophages, Schwann cells, and lepromatous leprosy lesions could be used *in vitro* to modulate *FOXP3* expression and Treg cell function in CD4 ^+^ T cells ^22– 24, 34, 36, 88, 89^.

## Conclusions

The data presented here suggest that modulation of host cell metabolism contributes to the maintenance of a functional program in infected macrophages and Schwann cells that suits *M. leprae* survival and proliferation, while it downregulates immune response against the pathogen, creating conditions for a chronic infection. The microenvironment generated particularly in the infected tissue of lepromatous leprosy also activates host disease tolerance mechanisms that allow these patients to display moderate pathology despite the high bacterial burden. A better understanding of the role of Treg cells in the suppression of Th1 response can have a major impact in leprosy control by the generation of new potential intervention tools, particularly in the population of exposed individuals with asymptomatic disease. Dissecting the metabolic pathways that favor Treg cell generation modulated by *M. leprae* infection may contribute to the identification of biomarkers for the early disease diagnosis. Most importantly, new pharmacological targets that block the inhibition of IFN-γ production would potentially reduce the number of individuals evolving to active disease or at least the frequency of lepromatous leprosy that significantly impacts leprosy transmission. In this context, non-steroidal anti-inflammatory drugs, which are COX-2 inhibitors, could at the same time lower PGE _2_ levels and IDO1 expression, leading to Treg cell downmodulation as seen in the reversion of tumor immune evasion in cancer models ^53, 90^. Furthermore, these drugs are widely available at low cost, which is an important point given the lack of resources for neglected diseases.
